# Comparative Study on Synergistic Toxicity of Enrofloxacin Combined with Three Antibiotics on Proliferation of THLE-2 Cell

**DOI:** 10.3390/antibiotics11030394

**Published:** 2022-03-16

**Authors:** Yehui Luan, Kexin Chen, Junjie Zhao, Linli Cheng

**Affiliations:** College of Veterinary Medicine, China Agricultural University, Beijing 100193, China; pharma2@cau.edu.cn (Y.L.); chengsijia@cau.edu.cn (K.C.); jinzelv@cau.edu.cn (J.Z.)

**Keywords:** synergistic toxicity, enrofloxacin, ciprofloxacin, florfenicol, sulfadimidine, binary combination, proliferation, comparative study

## Abstract

Little attention has been paid to the problem of the combined toxicity of accumulated antibiotics on humans from food and clinical treatments. Therefore, we used human hepatocytes to study the joint toxicity of four common antibiotics. The cytotoxicity of enrofloxacin (ENR), combined with ciprofloxacin (CFX), florfenicol (FFC), or sulfadimidine (SMD) on THLE-2 cells was determined by CCK-8 assays; then their joint toxicity was evaluated using CalcuSyn 2.0. Dose–effect curves and median-effect plots established on large amounts of data and CI values were calculated to judge the nature of the combination’s interaction. ED50, ED75, and ED90 were predicted to elucidate the changing trend of the concentration on the toxicity of each drug pair. The ENR-CFX and ENR-FFC pairs exhibited synergistic toxicity only at special concentration rates, while ENR and SMD synergistically induced cytotoxicity at almost all the concentration rates studied. The mixed ratio was a significant factor for synergistic toxicity and should be evaluated in all combined effect studies. These results suggested that the combined toxicity of these four drugs should be taken into account in their risk assessment.

## 1. Introduction

In recent years, there has been a growing awareness of the combined toxicity of multiple residual drugs caused by mixed accumulates in food and the environment [1,2,3,4]. Until now, almost all chemical risk evaluations were established by the research of single compounds [5,6]. The European Food Safety Authority (EFSA) proposed concerns about the joint toxicity of drugs in 2006, and after thirteen years of preparation, the risk assessment of the joint toxicity of pesticides was finally initiated [7,8]. According to current EU considerations, joint toxicity studies were published on insecticides, fungicides, and other chemicals [9,10,11]. However, the joint toxicity of residual antibiotics remained largely unmentioned and was not taken into account in their regulation and safety assessment. Therefore, we investigate the joint toxicities of some commonly used antibiotics with cell models and statistical methods.

Enrofloxacin, ciprofloxacin, florfenicol, and sulfadimidine were selected as research targets, as they are widely used antimicrobials in the animal breeding industry and in humans [12]. All of the selected antimicrobials have been directly detected in fresh foods such as meat, eggs, and even vegetables [13,14], and were found to spread through the environment and, in turn, adversely impact human health [15,16]. The combined microbial residues may affect humans.

Enrofloxacin, ciprofloxacin, and florfenicol have immunosuppressive activities [17,18,19,20]. Florfenicol can interfere with liver and renal functions and disrupt the intestinal mucosal barrier [19,20,21]. High doses of sulfadimidine are associated with a significantly increased incidence of thyroid tumors in mice and rats [22]. Toxicology and pharmacokinetics of the four antimicrobials were referenced in the WTO, EU, and China, laying out their MRL standards in animal origin foods (Table 1) [23,24,25]. However, if we consider the combined toxicity of these drugs, it may be necessary to take a second look at these MRLs. Considering that the liver is the site of metabolism for most drugs, including these four [26], we proposed using a liver cell model to examine their binary combined toxicity to cell multiplication.

## 2. Results and Discussion

### 2.1. Single Drug Toxicity

The CCK-8 assay was performed on THLE-2 hepatocytes to determine the cytotoxicity of enrofloxacin (ENR), ciprofloxacin (CFX), florfenicol (FFC), and sulfadimidine (SMD). All the drugs exhibited a dose-dependent inhibition (Table 2). The Dm values of ENR, CFX, FFC, and SMD were 13.11, 32.03, 392.5, and 358.6, respectively. ENR exhibited the greatest toxicity, with an inhibitory ratio of 58.78:84.25 within the dose range of 25 μg·L^−1^ to 500 μg·L^−1^. CFX revealed much higher toxicity than FFC and SMD at the same concentrations. These three drugs had an inhibitory ratio of 41.35:85.84, 17.93:54.75, and 18.39:54.55 within the dose range of 25 μg·L^−1^ to 500 μg·L^−1^, respectively.

**Figure 1:**
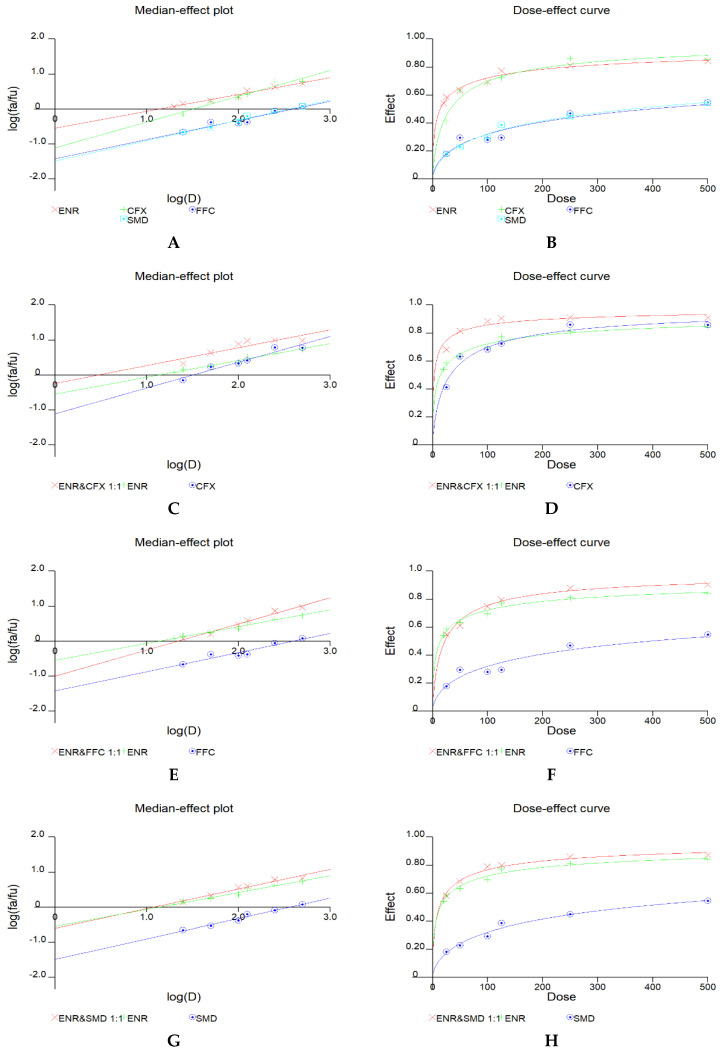
Median-effect plots and dose–effect curves of single drugs (**A**,**B**) and binary drug combinations mixed at a ratio of 1:1 (**C**–**H**).

The CalcuSyn2.0 software was used to generate dose–effect curves and median-effect plots for single drugs in Figure 1A,B. All the (r) values of the median-effect plots were above 0.95, demonstrating that the experimental data agreed well with the median-effect equation of Chou. The dose–effect curves and median-effect plots did not fit well at very low concentrations since antimicrobials promote growth at low concentrations. The dose–effect curves of all the drugs had a flat sigmoidal shape (M < 1). Dm affords the toxic potency on THLE-2 cells, ENR > CFX > FFC > SMD. It was interesting that the ENR and CFX showed a cross point at a dose of about 180 µg·L^−1^, which was near their MRLs in poultry liver (200 µg·kg^−1^). The toxicity expression varied below and above this MRL for this two-drug combination, with the same primary mechanism, suggesting that they must have a different secondary toxicity mechanism.

### 2.2. Joint Toxicity of Three Binary Drug Combinations

The combined toxicity of ENR-CFX, ENR-FFC, and ENR-SMD was calculated. The drugs were initially mixed at the concentration ratio of 1:1 in six different dose groups. CalcuSyn2.0 software was again used to calculate the dose–effect curves, median-effect plots, and CI values for the binary combination in Figure 1C–H. All the curves correspond well with the median-effect equation of Chou with a correlation rate of over 0.88.

CI values varied at different concentrations on THLE-2 cells, as shown in Table 3. The joint toxicity of ENR-CFX showed synergism over certain dose ranges, and their CI values ranged from 0.264 to 0.651 within the dose range of (25,25) to (250,250) µg·L^−1^. The joint toxicity of ENR-FFC was mutually enhanced at high concentrations, with CI values ranging from 0.383 to 0.831 within the dose range of (100,100) to (500,500) µg·L^−1^. The joint toxicity of ENR-SMD exhibited synergism at each dose, except (25,25) µg·L^−1^. The CI values of ENR-SMD ranged from 0.453 to 1.003. The dose–effect curves of all binary combinations showed a flat sigmoidal shape (M < 1). We conclude that on THLE-2 cells, ENR-CFX, ENR-FFC, and ENR-SMD exhibited dose-dependent synergistic toxicity, and the synergistic toxicity of ENR-SMD was the most notable.

**Figure 2:**
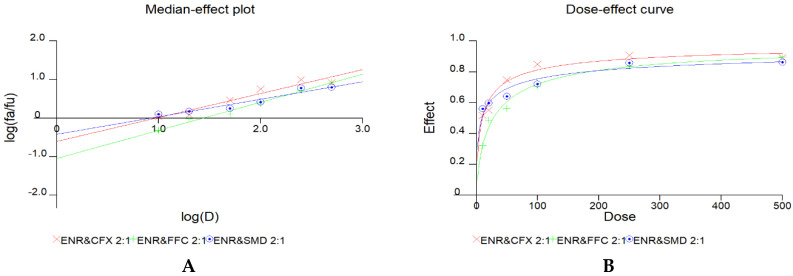

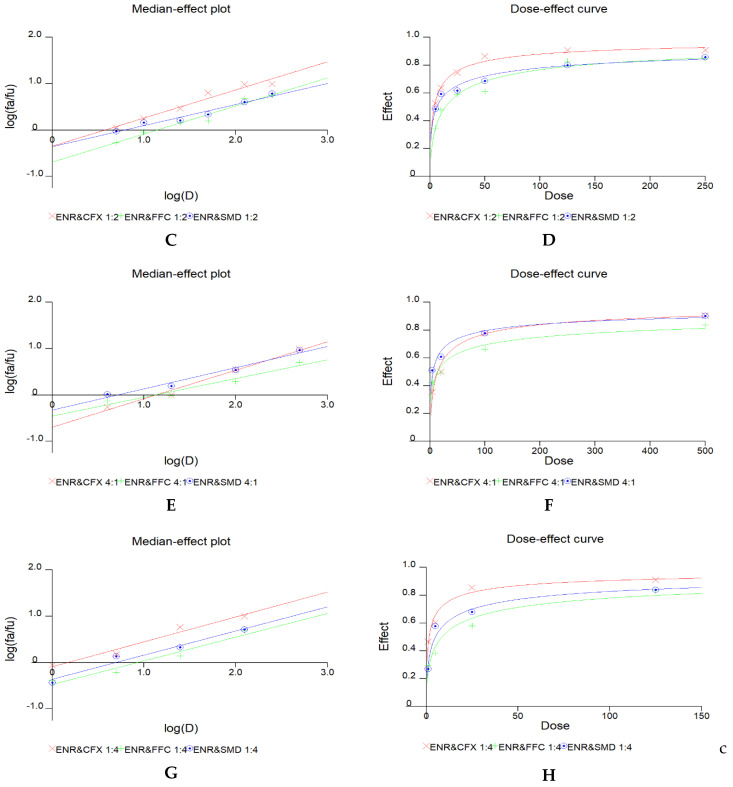
A caption on a single line should be centered. Median-effect plots and dose–effect curves of binary drug combinations mixed at a ratio of 1:2 (**A**,**B**), 2:1 (**C**,**D**), 4:1 (**E**,**F**), and 1:4 (**G**,**H**).

Then, we determined whether the mixing ratio impacted joint toxicity. Three binary combinations were mixed at the ratios of 1:2, 1:4, 2:1, and 4:1 for at least four concentrations. Similarly, we performed another CCK-8 assay and calculated the CI value for each group via CalcuSyn software. The results are presented in Table 4, Table 5 and Table 6, and Figure 2.

A significant difference was observed in some drug combinations and mixing ratios. When the mixing ratio was 2:1 and 4:1, the joint effect of ENR-CFX could either exhibit synergism or antagonism. In contrast, when the mixing ratio was 1:2 and 1:4, their joint effect showed only synergism. In addition, for the binary combination ENR-SMD, their joint toxicity showed the strongest synergism when the mixing ratio was 1:4, but not 1:1 at the concentration of (5,20) µg·L^−1^ with the CI value of 0.245.

**Figure 3:**
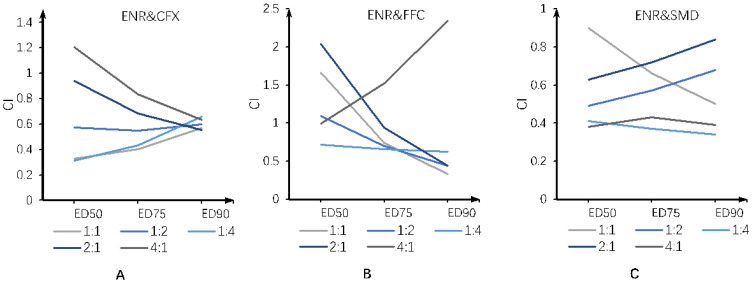
Comparison of CI values of binary combination enrofloxacin and ciprofloxacin (**A**), enrofloxacin and florfenicol (**B**), and enrofloxacin and sulfamethazine (**C**) at different mixing ratios.

We sought to compare their CI values with the same effect to visualize the joint toxicity difference of each mixing ratio. The CI values of each mixing ratio were predicted at ED50, ED75, and ED90, respectively, using the previously obtained median-effect plots (Figure 3).

The synergistic toxicity for ENR-CFX was strongest at the 1:1 ratio, but weakest at the 4:1 ratio. The synergistic toxicity for ENR-FFC was the strongest at a ratio of 1:4 with an ED50, while at ED75 and ED90, the synergistic toxicities of ENR-FFC at 1:4 1:2, 1:1, and 2:1 were similar. The synergistic toxicity for ENR-SMD was stronger at 1:4 and 4:1 compared to other mixing ratios. Thus, we demonstrated that the joint toxicity of binary drug mixtures is mixing-ratio-dependent.

Previous studies on joint toxicity typically used a 1:1 mixing ratio, but our results argued that an experimental design with a single mixing ratio is inappropriate. The clinical dosage of drugs in this study was similar; thus, their residue levels in food are roughly the same. However, during actual use, there may be situations in which the dosage is privately changed, resulting in different residual proportions of drugs in food. Therefore, a pairwise testing of 1:1, 1:2, 1:4, 2:1, and 4:1 mixing ratios or other combinations of drugs is required to obtain proper results. ENR and CFX are frequently used in animals, and their chemical structures are similar; furthermore, the former can be metabolized in vivo to the latter [13]. As a result, ENR and CFX were recognized as drugs with a similar mode of action. ENR and CFX reportedly inhibit CYP450 enzymes, which are responsible for drug metabolism in liver cells [27]. The inhibition of CYP450 may be a possible mechanism for the cytotoxicity of these drugs. However, combining drugs with identical mechanisms can only lead to addictive effects or antagonism. The synergistic toxicity of ENR-CFX indicates that ENR potentially has a different toxic mechanism from that of CFX. FFC was recently shown to induce noticeable cytotoxicity by inhibiting mitochondrial protein synthesis [28]. These two cytotoxicity mechanisms may combine, leading to synergistic toxicity. The mechanisms of combined toxicity are still uncertain and require further studies.

The median-effect equation of Chou is derived based on enzyme kinetic models of the law of mass-action, widely recognized in the field of medicine [29]. One of the advantages of the Chou–Talalay method is that it does not require many experiments. For each mixing ratio of each drug combination, four to seven determining concentrations are sufficient for fitting median-effect plots. Data for calculating CI values at each Fa can be acquired by implementing a coefficient simulation, greatly reducing experimental costs. The Chou–Talalay method provides two Formulas (2) and (3) useful for calculating drugs with the same or different modes of action. However, there is generally no significant difference between the computed results of these two formulas. Since synergistic joint toxicity results are more useful to food safety risk assessment, ENR-FFC and ENR-SMD were treated as mutually non-exclusive drugs to ensure that CI < 1 indicates synergistic joint toxicity.

Organisms can be enriched by veterinary antimicrobials in the environment [30]. As the detection technology developed, several residual antimicrobials in fresh food can be detected using one-time testing [14]. Knowing the joint effects helps in food safety assessment, yet toxicology research for veterinary antimicrobials still focuses on single drugs. Taking enrofloxacin as an example, our study shows that its potential toxicity in fresh food is affected by other antimicrobials. Enrofloxacin’s toxicity is enhanced when combined compared to the toxicity from each drug residue alone—whether the drugs have the same or different mechanisms. Therefore, it is important to establish a database of joint toxicities of veterinary drugs. The original MRLs need to be adjusted, or a new MRL evaluating standard needs to be developed in which drug interactions are considered. In our opinion, it is important to evaluate and refine existing methodologies for assessing risks of exposure to two or more veterinary antimicrobials in combination, particularly in the context of setting MRLs in accord with government regulations such as EC 396/2005. Ideally, risk assessments in veterinary medicine should consider all possible residues (e.g., individually or in different ratios of combination) that influence pathways (e.g., fresh food, processed food, feeds) and routes of exposure (e.g., ingestion, dermal, inhalation) contributing to total exposure. However, appropriate data on levels of exposure in veterinary medicine from pathways and sources are not generally available, and further research is required. Therefore, the actual MRLs still need to be discussed while fresh foods face multiple sources of pollution of veterinary antimicrobials.

Nevertheless, the method in this study has limitations, such as it cannot evaluate the joint toxic effects that are difficult to quantify, such as neurotoxicity. Other approaches, such as animal studies, are required in this case.

## 3. Materials and Methods

Enrofloxacin was selected as the main drug in three binary drug combinations: enrofloxacin and ciprofloxacin, enrofloxacin and florfenicol, and enrofloxacin and sulfadimidine. Three different concentrations (high, medium, and low) for each of the four drugs were set based on their MRL values in the animal liver for pairwise testing (Table 1). The MRL of sulfadimidine was used as the lowest concentration of all drugs for calculation convenience, as it is the lowest MRL of the four antimicrobials. Hence, nine preparations were generated for each binary drug combination 1:1 (low-low, medium-medium, high-high), 1:2 (medium-high, low-medium), 1:4 (low-high), 2:1 (high-medium, low-medium), and 4:1 (high-low). Counting the single drug and blank controls, there were a total of 40 preparations for the three binary drug combinations. We performed three five-fold serial dilutions on all 40 preparations to obtain sufficient data for linear fitting.

THLE-2 immortalized human hepatocytes were provided by the Institute of Biochemistry and Cell Biology, Chinese Academy of Sciences (Shanghai, China) and were cultivated at 37 °C with 5% CO_2_ in the BEGM Bullet Kit (CC-3170) from Lonza, which includes 500 mL basal medium and separate frozen additives. The gentamycin/amphotericin (GA) and epinephrine were eliminated. An extra 6 ng/mL human recombinant EGF (Sigma Aldrich, St. Louis, MO, USA), 80 ng·mL^−1^ phosphoethanolamine (Sigma Aldrich), and 10% fetal bovine serum were added as the final growth medium. The coating medium was set as the RPMI1640 without glutamine supplementation with 0.01 mg·mL^−1^ bovine serum albumin (heat shock fraction, Sigma), 0.03 mg·mL^−1^ type I collagen from bovine skin (Sigma), and 0.01 mg·mL^−1^ fibronectin from human plasma (Sigma). An atmosphere of 95% air and 5% carbon dioxide (CO_2_) was used for culturing, and the temperature was 37.0 °C. The flasks and plates used were pre-coated with a mixture of 0.01 mg·mL^−1^ fibronectin, 0.03 mg·mL^−1^ bovine collagen type I, and 0.01 mg·mL^−1^ bovine serum albumin dissolved in BEBM medium. The sub-cultivation ratio was from 1:6 to 1:4. Every 2 to 3 days, the medium was renewed. Complete growth medium supplemented with 5% (*v/v*) DMSO as the freezing medium, and cells were stored under a liquid nitrogen vapor phase.

A CCK-8 assay was used to detect cytotoxicity for single drugs and binary drug mixtures. Enrofloxacin, ciprofloxacin, florfenicol, sulfadimidine, and their binary mixtures were dissolved in DMSO at a set concentration as a working solution. One day before the assay, cells were cultivated at 500 cells/well in 384-well plates where the original medium was replaced by a working-fluid-containing medium on the day of the assay. The incubation was performed for 72 h at a 37 °C, 5% CO_2_ incubator. Subsequently, CCK-8 was added to each well, and the plates were incubated for another 2 h. Determination of absorbance at 450 nm wavelength was performed by a microplate reader (PerkinElmer VICTOR Nivo), and the optical density (OD) value of each well was obtained. Finally, the inhibition ratio was measured as the cytotoxic effect using the following Formula (1).

(1) $$\mathrm{Inhibition}\;\mathrm{rate}\;\left(\mathrm{IR}\right)=\left[1-\frac{\left({\left[\mathrm{OD}\right]}_{\mathrm{treated}}-{\left[\mathrm{OD}\right]}_{\mathrm{blank}}\right)}{\left({\left[\mathrm{OD}\right]}_{\mathrm{control}}-{\left[\mathrm{OD}\right]}_{\mathrm{blank}}\right)}\right]\times 100\%$$

where [OD]_treated_ represents the mean absorbance of the cells treated with working fluid, [OD]_control_ represents the mean absorbance of the cells treated with the mixture of DMSO and cultural medium, and the [OD]_blank_ corresponds to the blank control.

The Chou–Talalay Method [27] was used to assess the drug interaction in the cell model after we arranged the original data in Microsoft Excel. The invalid data whose values fluctuated narrowly around zero were eliminated before data analysis. The median-effect plot for each single drug was first calculated to measure the combination index (CI) value based on the Median-Effect Equation of Chou. For drugs with the same mode of actions, CI values were calculated according to Formula (2). For drugs with the different mode of actions, CI values were calculated according to Formular (3).

(2) $$\mathrm{CI}\left(x\right)=\frac{{\left(D\right)}_{1}}{{\left(D_{x}\right)}_{1}}+\frac{{\left(D\right)}_{2}}{{\left(D_{x}\right)}_{2}}$$

(3) $$\mathrm{CI}\left(x\right)=\frac{{\left(D\right)}_{1}}{{\left(D_{x}\right)}_{1}}+\frac{{\left(D\right)}_{2}}{{\left(D_{x}\right)}_{2}}+\frac{{\left(D\right)}_{1}{\left(D\right)}_{2}}{{\left(D_{x}\right)}_{1}{\left(D_{x}\right)}_{2}}$$

where, D_1_, D_2_ are the doses of each drug in the binary combination that exhibits an x inhibition, D_x_ is the dose of the single drug required that exhibits an x inhibition alone.

## 4. Conclusions

We evaluated the toxicity of four common veterinary drugs and performed the Chou–Talalay method to evaluate the joint toxicity of three binary combinations. These studies revealed that on THLE-2 cells, the inhibitory ratio of ENR and CFX was higher than that of FFC and SMD. Furthermore, combining ENR with CFX, ENR with FFC, and ENR with SMD synergistically induced cytotoxicity in dose-independent and mixing-ratio-independent conditions. We also showed that in a joint toxicity experiment, different concentration ratios are required to assess combined drugs. The preferred ratios were 1:1, 1:2, 1:4, 2:1, and 4:1 in pairwise testing equipment. Our results illustrated that new assessments for food safety should consider combined exposure and toxicity. The concentration settings also need to be considered when determining the effect of both mixing ratio and drug type on the combined toxicity.

## Figures and Tables

**Table 1:** Maximum residual limits (MRL) of the compounds tested.

Animal	Tissues	Enrofloxacin, Ciprofloxacin			Sulfadimidine			Florfenicol		
		Sum of Enrofloxacin and Ciprofloxacin (µg·kg^−1^)			Sulfadimidine (µg·kg^−1^)			Sum of Florfenicol and Florfenicol-Amine (µg·kg^−1^)		
		WHO	EU	China	WHO	EU	China	WHO	EU	China
ADI		0–6.2				0–50			0–3	
Cattle/Sheep	Muscle	100	100	100	100	100	100	200	200	200
	Fat	100	100	100	100	100	100	-	-	-
	Liver	300	300	300	100	100	100	3000	3000	3000
	Kidney	200	200	200	100	100	100	300	300	300
	Milk	100	100	100	25				-	-
Pig/Rabbit	Muscle	100	100	100	100	100	100	300	300	300
	Fat	100	100	100	100	100	100	500	500	500
	Liver	200	200	200	100	100	100	2000	2000	2000
	Kidney	300	300	300	100	100	100	500	500	500
Poultry (Prohibit in laying period)	Muscle	100	100	100	100	100	100	100	100	100
	Skin + fat	100	100	100	100	100	100	200	200	200
	Liver	200	200	200	100	100	100	2500	2500	2500
	Kidney	300	300	300	100	100	100	750	750	750
Other	Muscle	100	100	100	100	100	100	100	100	100
	Fat	100	100	100	-		100	200	200	200
	Liver	-	-	200	-		100	2000	2000	2000
	Kidney		-		-		100	300	300	300
Fish	Skin + Fat	100			-		100	1000	1000	1000

**Table 2:** Parameters of median-effect plots of enrofloxacin (ENR), ciprofloxacin (CFX), florfenicol (FFC), and sulfamethazine (SMD) for THLE-2 cells after 72 h incubation.

Drugs	D/μg·L^−1^	Fa	M	Dm	r
ENR	25	0.5878	0.4734	13.11	0.979
	50	0.6353			
	100	0.6949			
	125	0.7726			
	250	0.8108			
	500	0.8425			
CFX	25	0.4135	0.7273	32.03	0.967
	50	0.6324			
	100	0.6830			
	125	0.7234			
	250	0.8604			
	500	0.8584			
FFC	25	0.1793	0.5460	392.5	0.951
	50	0.2970			
	100	0.2804			
	125	0.2969			
	250	0.4704			
	500	0.5475			
SMD	25	0.1839	0.5804	358.6	0.988
	50	0.2284			
	100	0.2948			
	125	0.3866			
	250	0.4501			
	500	0.5455			

D, drug dose; Fa, fraction affected by dose; M, shape parameter; r, linear correlation coefficient; Dm, median-effect drug dose.

**Table 3:** CI values and parameters of median-effect plots of binary drug combinations (mixed at 1:1) against THLE-2 cells after 72 h incubation.

Drugs				Parameters				
ENR/μg·L^−1^	CFX/μg·L^−1^	FFC/μg·L^−1^	SMD/μg·L^−1^	Fa	M	Dm	r	CI
25	25			0.6825	0.5097	2.940	0.889	0.760
50	50			0.8142				0.414
100	100			0.8850				0.319
125	125			0.9057				0.277
250	250			0.9092				0.580
500	500			0.9073				1.234
25		25		0.5470	0.7452	21.79	0.987	1.289
50		50		0.6106				1.489
100		100		0.7472				0.804
125		125		0.7974				0.555
250		250		0.8800				0.306
500		500		0.9027				0.375
25			25	0.5817	0.5598	11.82	0.980	0.966
50			50	0.6815				0.792
100			100	0.7906				0.490
125			125	0.7990				0.550
250			250	0.8610				0.441
500			500	0.8727				0.716

Fa, fraction affected by dose; M, shape parameter; Dm, median-effect drug dose; CI, combination index.

**Table 4:** CI values and parameters of median-effect plots of binary drug combination enrofloxacin and ciprofloxacin (mixed at ratios of 2:1, 4:1, 1:2, and 1:4) against THLE-2 cells after 72 h incubation.

Mixing Ratio	ENR/μg·L^−1^	CFX/μg·L^−1^	Fa	M	Dm	r	CI
2:1	10	5	0.5180	0.6196	9.373	0.964	0.867
	20	10	0.5518				1.418
	50	25	0.7472				0.635
	100	50	0.8502				0.372
	250	125	0.9080				0.352
	500	250	0.8931				1.058
4:1	4	1	0.3554	0.6127	13.51	0.991	1.163
	20	5	0.4999				1.921
	100	25	0.7783				0.756
	500	125	0.9066				0.548
1:2	5	10	0.5270	0.6057	3.737	0.979	0.645
	10	20	0.6370				0.588
	25	50	0.7454				0.633
	50	100	0.8641				0.350
	125	250	0.9069				0.462
	250	500	0.9085				0.947
1:4	1	4	0.4683	0.5365	1.463	0.983	0.259
	5	20	0.6033				0.567
	25	100	0.8547				0.340
	125	500	0.9089				0.814

Fa, fraction affected by dose; M, shape parameter; Dm, median-effect drug dose (represented as the concentration of enrofloxacin (μg·L^−1^)); CI, combination index.

**Table 5:** CI values and parameters of median-effect plots of binary drug combination enrofloxacin and florfenicol (mixed at ratios of 2:1, 4:1, 1:2, and 1:4) against THLE-2 cells after 72 h incubation.

Mixing Ratio	ENR/μg·L^−1^	FFC/μg·L^−1^	Fa	M	Dm	r	CI
2:1	10	5	0.3228	0.7269	27.26	0.994	3.506
	20	10	0.4876				1.663
	50	25	0.5619				2.233
	100	50	0.7071				1.198
	250	125	0.8390				0.605
	500	250	0.8973				0.413
4:1	4	1	0.4245	0.4040	13.26	0.973	0.561
	20	5	0.4917				1.593
	100	25	0.6625				1.824
	500	125	0.8358				1.254
1:2	5	10	0.3483	0.6045	13.89	0.979	1.441
	10	20	0.4786				0.941
	25	50	0.5913				0.939
	50	100	0.6107				1.553
	125	250	0.8262				0.393
	250	500	0.8484				0.562
1:4	1	4	0.2944	0.5110	8.434	0.973	0.507
	5	20	0.3830				1.123
	25	100	0.5813				1.088
	125	500	0.8319				0.417

Fa, fraction affected by dose; M, shape parameter; Dm, median-effect drug dose (represented as the concentration of enrofloxacin (μg·L^−1^)); CI, combination index.

**Table 6:** CI values and parameters of median-effect plots of binary drug combination enrofloxacin and sulfamethazine (C) (at ratios of 2:1, 4:1, 1:2, and 1:4) against THLE-2 cells after 72 h incubation.

Mixing Ratio	ENR/μg·L^−1^	FFC/μg·L^−1^	Fa	M	Dm	r	CI
2:1	10	5	0.5610	0.4528	8.336	0.964	0.451
	20	10	0.5980				0.658
	50	25	0.6405				1.131
	100	50	0.7217				1.037
	250	125	0.8573				0.454
	500	250	0.8633				0.818
4:1	4	1	0.5099	0.4570	5.145	0.985	0.274
	20	5	0.6098				0.592
	100	25	0.7769				0.554
	500	125	0.9023				0.365
1:2	5	10	0.4848	0.4537	6.124	0.980	0.449
	10	20	0.5934				0.364
	25	50	0.6182				0.735
	50	100	0.686				0.795
	125	250	0.8001				0.575
	250	500	0.8588				0.489
1:4	1	4	0.2707	0.5191	4.872	0.982	0.644
	5	20	0.5776				0.224
	25	100	0.6808				0.455
	125	500	0.8383				0.380

Fa, fraction affected by dose; M, shape parameter; Dm, median-effect drug dose (represented as the concentration of enrofloxacin (μg·L^−1^)); CI, combination index.

## Data Availability

Not applicable.
